# Cytochrome b sequence of the *Mazama americana jucunda* Thomas, 1913 holotype reveals *Mazama bororo* Duarte, 1996 as its junior synonym

**DOI:** 10.1590/1678-4685-GMB-2021-0093

**Published:** 2021-12-13

**Authors:** Aline Meira Bonfim Mantellatto, Susana González, José Maurício Barbanti Duarte

**Affiliations:** 1 Universidade Estadual Paulista (UNESP), Faculdade de Ciências Agrárias e Veterinárias, Núcleo de Pesquisa e Conservação de Cervídeos, Jaboticabal, SP, Brazil.; 2 Instituto de Investigaciones Biológicas Clemente Estable, Departamento de Biodiversidad y Genética, Montevideo, Uruguay.

**Keywords:** Cytochrome b, small red brocket deer, taxonomy, phylogenetic analysis, Atlantic Forest

## Abstract

The small red brocket deer, *Mazama bororo* Duarte, 1996 was described based on karyotypical and morphological characters. However, the original description of *Mazama americana jucunda* suggested that this subspecies could represent the same taxon as *Mazama bororo*. This assumption was based on the type locality of *Mazama americana jucunda* and on morphological similarities between *Mazama americana jucunda* and *Mazama bororo*. To solve this question, we obtained DNA sequences of the holotype of *Mazama americana jucunda* and compared it with other species of *Mazama*, including the holotype of *M. bororo*. A phylogenetic tree was obtained to verify the relationships among these taxa. The results clearly showed that *M. americana jucunda* and *M. bororo* represent the same biological entity. Therefore, the oldest name available for the small red brocket occurring in the Atlantic Forest of southern Brazil should be *Mazama jucunda*, remaining *M. bororo* as a junior synonym. We emphasise the importance of using DNA from museum specimens, especially from holotypes, in order to obtain a more accurate taxonomic identification. We also highlight the importance of application of valid names for labelling all aspects of biodiversity research, as well as for monitoring and conservation efforts.

Natural history collections are unique repositories of biodiversity, offering great opportunities for genetic research that can be applied in taxonomy and conservation (Burrel *et al.,* 2015). The number of taxonomic studies using DNA obtained from specimens deposited in natural history collections has increased mainly due to the use of the mitochondrial genes (Hajibabaei et al., 2007). Such availability of sequences obtained from type specimens can provide additional data for poorly known taxa, which facilitates the description of new species and taxonomic revisions (Chakrabarty, 2010; Strutzenberger et al., 2012). 

The existence of several cryptic species of *Mazama* is considered one of the more impressive striking case of morphological convergence within mammals (Gilbert et al., 2006), remaining doubts to analyse the evolutionary relationships (Duarte et al., 2008). The monophyly of this genus was refuted by molecular analyses using mitochondrial and nuclear loci (Gilbert *et al.,* 2006; Duarte *et al.,* 2008; Gutiérrez et al., 2017).

The small red brocket deer, *Mazama bororo*, was proposed by Duarte in 1996 based on morphological (Duarte, 1996) and cytogenetic characterisation (Duarte and Giannoni, 1996; Duarte and Jorge, 2003). This classification was predominantly based on karyotype differences, as *M. bororo* had a completely different chromosomal pattern, suggesting its probable reproductive isolation from other *Mazama* (Vogliotti and Duarte, 2012). External body measurements of *M. bororo* were intermediate between *M. americana* and *M. nana*, and very close to those of hybrids between the latter two species (Duarte and Jorge, 2003). The hybrids between *M. americana* and *M. nana*, although morphologically similar to *M. bororo*, have the chromosomal set of both parents and, therefore, are easily distinguishable by cytogenetic analyses (Vogliotti and Duarte, 2012).

Morphological studies of *Mazama* specimens based on skeletons and skins from different Brazilian collections did not detect significant differences among *M. bororo* from *M. americana* (Vogliotti and Duarte, 2010). However, specific traits are potentially discriminant for the species, including weight, height, body length, thorax circumference, and the lengths of the metacarpus and metatarsus (Duarte and Jorge, 2003), at least in living animals or recently decreased specimens (Vogliotti and Duarte, 2010).

The name *Mazama americana jucunda* Thomas, 1913 is the oldest taxon of *Mazama* with the type locality in Brazil. The type specimen was collected in 1901, in the region of Roça Nova, on the Serra do Mar, state of Paraná, Brazil, in a region where currently inhabits *M. bororo*. In addition, the morphological description closely resembles the characteristics of *M. bororo*, as described by Duarte (1996). Thomas described *M. a. jucunda* as smaller than the other known species of *Mazama,* with the upper part of the limbs showing a reddish-brown colour on the metacarpals, and a darker tail in the dorsal region. Considering that the taxon described as *M. bororo* may be conspecific to *M. a. jucunda*, we used mitochondrial DNA sequences from the holotypes to investigate the possible synonymy between these two taxa. 

A total of 38 specimens of *Mazama* and one of *Ozotoceros bezoarticus* were analysed for genetic comparisons. We used 28 DNA sequences available on Genbank and 11 DNA sequences produced by this work also deposited in GenBank (Table 1). We extracted DNA samples from hairs of nine specimens of *M. bororo*, one of the *M. a. jucunda* holotype and one specimen of *O. bezoarticus* which was stored at *Núcleo de Pesquisa e Conservação de Cervídeos* (NUPECCE) tissue and cell bank. All samples were collected at a maximum of 30 years ago, except for the *M. a. jucunda,* which was collected 119 years ago. To extract DNA from *M. a. jucunda* we used a small fragment from a skull provided by the Natural History Museum (BMNH), London (specimen BMNH 3.7.1.103).

**Table 1 -:** Mitochondrial cytochrome *b* sequences used for phylogenetic inference among several *Mazama* specimens.

Species	Sample identification	GenBank Access	Origin/Source
*Mazama americana*	T16	DQ789209.2	Cuiabá-MT. Brazil/Captivity
	T18	DQ789211.2	Vilhena-RO. Brazil/Captivity
	T21	DQ789216.2	Ariquemes-RO. Brazil/Captivity
	T22	DQ789217.2	Ariquemes-RO. Brazil/Captivity
	T28	DQ789218.2	Rio Branco-AC. Brazil/Captivity
	T35^1^	DQ789221.2	Belém Zoo-AM. Brazil/Captivity
	T36^1^	DQ789222.2	Projeto Jari-PA. Brazil/Captivity
	T39	DQ789223.2	Parauapebas-PR.Brazil/Captivity
	T40^2^	DQ789224.2	Carajás-PA. Brazil/Captivity
	T41	DQ789225.2	Carajás-PA. Brazil/Captivity
	T43	MG786262	Carajás-PA. Brazil/Captivity
	T70^3^	DQ789230.2	Ciudad del Este.Paraguay/Captivity
	T110^3^	DQ789201.2	Terra Boa-PR. Brazil/Captivity
	T120^3^	DQ789204.2	Unknown/Captivity
	T161^4^	DQ789207.2	Carajás-PA. Brazil/Captivity
	T164^4^	DQ789208.2	Carajás-PA. Brazil/Captivity
	T192^2^	DQ789212.2	Unknown/Captivity
	T205	DQ789215.2	Foz do Iguaçu-PR. Brazil/Captivity
	T358	MN726911	Reginá. French Guiana/Wild
*Mazama bororo*	T64	DQ789228.2	Curitiba-PR. Brazil/Wild
	Msg54^5^	DQ789187.2	São Paulo-SP. Brazil/Captivity
	T71^5^	DQ789231.2	Barra do Turvo-PR. Brazil/Wild
	T72	MG786263.1	Barra do Turvo-PR. Brazil/Wild
	T213^*^	MH593529	Paraná-PR. Brazil/Wild
	T332^5*^	MH593530	Paraná-PR. Brazil/Captivity
	T333^5*^	MH593531	Paraná-PR. Brazil/Captivity
	T334^5*^	MH593532	Paraná-PR -PR. Brazil/Captivity
	T335^5*^	MH593533	Paraná-PR. Brazil/Captivity
	T336^5*^	MH593534	Paraná-PR. Brazil/Captivity
	T337^5*^	MH593535	Paraná-PR. Brazil/Captivity
	T338^5^	MG786261	Paraná-PR. Brazil/Wild
	T340^5*^	MH593536	Paraná-PR. Brazil/Wild
*Mazama nana*	T2	DQ789214.2	Iguazu. Paraguay/Captivity
	T53	DQ789227.2	Paraná-PR. Brazil/Captivity
	T185	DQ789210.2	Céu Azul-PR. Brazil/Captivity
*Mazama nemorivaga*	T149	DQ789206.2	Rondônia - RO. Brazil/Captivity
*Mazama gouazoubira*	Msg001	DQ789179.2	Minas. Uruguay/Captivity
*Ozotoceros bezoarticus*	Sg1623^*^	MH593537	Salto. Uruguay/Captivity
*Mazama americana jucunda*	BMNH 3.7.1.103^*^	MH593538	Paraná. Brazil/Wild

^1,2,3,4,5^ supra index indicates sequences belonging to the same haplotype. The equal numbers indicate identical haplotypes.

^*^ DNA sequences produced by this work and deposited at Genbank.

We extracted DNA from hair samples using the protocol described by Sambrook et al. (1989), where a 224 base pair (bp) fragment of the cytochrome b mitochondrial gene was amplified using the primer pair IDMAZ224L (5’ CATCCGACACAATAACAGCA 3’) and IDMAZH, (5’ TCCTACGAATGCTGTGGCTA 3’) described by González et al. (2009). 

Cytochrome b fragments from hair DNA samples were amplified in a conventional thermocycler (Biometra T One Thermocycler), and the amplification reaction was performed in a final volume of 25.0 μL, containing: 1 x ImmoMix™ (Bioline)*,* 0.3 μM of each primer, 0.3 μM of bovine serum albumin, 15 ng/μL of DNA and 7.6 μL of water. The polymerase chain reaction (PCR) amplification conditions were 95 °C for 5 min, 35 cycles at 95 °C for 1 min, 55 °C for 1 min, 72 °C for 1 min; and a final extension at 72 °C for 10 min. PCR products were visualised on 1% agarose gel to verify the success in amplification and checked for the size of the fragments based on the 1 kb plus DNA ladder marker (Invitrogen). 

DNA extraction from the *M. a. jucunda* holotype was performed following the protocol described by González et al. (2015), and also a 224 base pair (bp) fragment was amplified using the same primer pair described above. To minimise the risks of contamination and ensure the reliability of the results, negative controls were used in all DNA extractions and during the PCR, which was performed three different times, in two different laboratories. PCR amplification for the DNA sample from the BMNH collection was performed in a final volume of 20.0 μL, containing: 1 x SensiFAST*™* HRM kit, 0.8 μM of primer, 0.3 μL BSA, 10 ng/μL of DNA and 6.9 μL of water. The real-time thermocycler (Rotor-Gene, Corbet*™*) programming was: 95 °C for 2 min, 95°C for 5 s, 54 °C for 10 s (10 cycles), then 54 °C for 10 s (15 cycles), 53 °C for 10 s, and 72 °C for 20 s. After purifying the samples, according to the protocol described by Dorado-Pérez (2012), each of the amplified samples were sequenced individually with the same primers (forward and reverse) used in amplification in an Applied Biosystems 3730xL automated sequencer.

The quality of the sequences obtained was analysed visually and using the PHRED software, contained in Codon Code Aligner v. 6.0.2. Sequences with less than 50 base-pairs with PHRED 20 were excluded. Sequence alignment was performed by the PHRED Clustal W (Thompson et al., 1994), contained in BioEdit v. 7.2.5 (Hall, 1999). All the sequences used in this work were aligned and restricted to 224 bp cytb fragment. To infer the best nucleotide substation model for the dataset, the sequences were analysed in jModelTest v. 2.1.6 (Darriba et al., 2012) as implemented in the CIPRES Science Gateway (Miller et al., 2010). The criterion used to select the best model was the Bayes information criterion (BIC), and the Hasegawa Kishino and Yano (HKY) model + Gamma was selected. Sequences for the mitochondrial *Cyt-b* gene were obtained by 224 bp fragment. The software BEAST v. 1.8.1 (Drummond et al., 2012) was used to infer the tree based on Bayesian Inference, and the Markov chains were run for 25,000,000 generations; trees were sampled every 1000 generations. A 25% burn-in was adopted. Therefore, the first 6,250,000 generations (6250 trees) were discarded as burn-in, and posterior probability estimates of all model parameters were based on the remaining (18,751) trees. The convergence between races was verified using the software Tracer v.1.6 and only effective sample size (ESS) results higher than 200 were accepted. The resulting trees were condensed in the programme Tree Annotator and were visualised using the programme Fig Tree, v.1.3.1 (Rambaut, 2010). Sequences of samples belonging to the *M. americana*, *M. bororo*, *M. a. jucunda, M. gouazoubira*, *M. nana*, *M. nemorivaga* and *O. bezoarticus* were used for phylogenetic tree inference (Table 1). The sequence of *O. bezoarticus*, *M. gouazoubira* and *M. nemorivaga* were used to root the tree, because these species belonged to the subtribe Blastocerina, and the group of the red brocket deer (*M. americana*, *M. bororo,* and *M. nana*) belonged to the subtribe Odocoileina (Gutiérrez et al., 2017; Heckeberg, 2020).

As a result, we identified 25 different haplotypes of the 39 sampled individuals. Among the 13 sequences of *M. bororo* analysed, we found four different haplotypes, and among the 19 sequences of *M. americana* analysed, we found 14 different haplotypes. For the others species (*M. nana*, *M. gouazoubira*, *M. nemorivaga* and *O. bezoarticus*) and subspecies (*M.a.jucunda*) were detected as unique haplotypes for each analysed sequence (Table 1). 

The Bayesian Inference analysis (Figure 1) showed that the sequence of the *M. a. jucunda* holotype was nested within the clade of *M. bororo* sequences and this clade showed high posterior probability support (= 1.0). A clear divergence was verified between *M. a. jucunda* and *M. americana* particularly considering the existence of *M. bororo*. The clades of *M. americana* and *M. bororo* are clearly separated by a posterior probability support (posterior probability of 0.95). Indeed, the fact of *M. a. jucunda* was not grouped on the *M. americana* clade highlighting that it may not be a subspecies of *M. americana*. Our result suggests that *M. a. jucunda* and *M. bororo* represent the same biological entity. 

**Figure 1 -:**
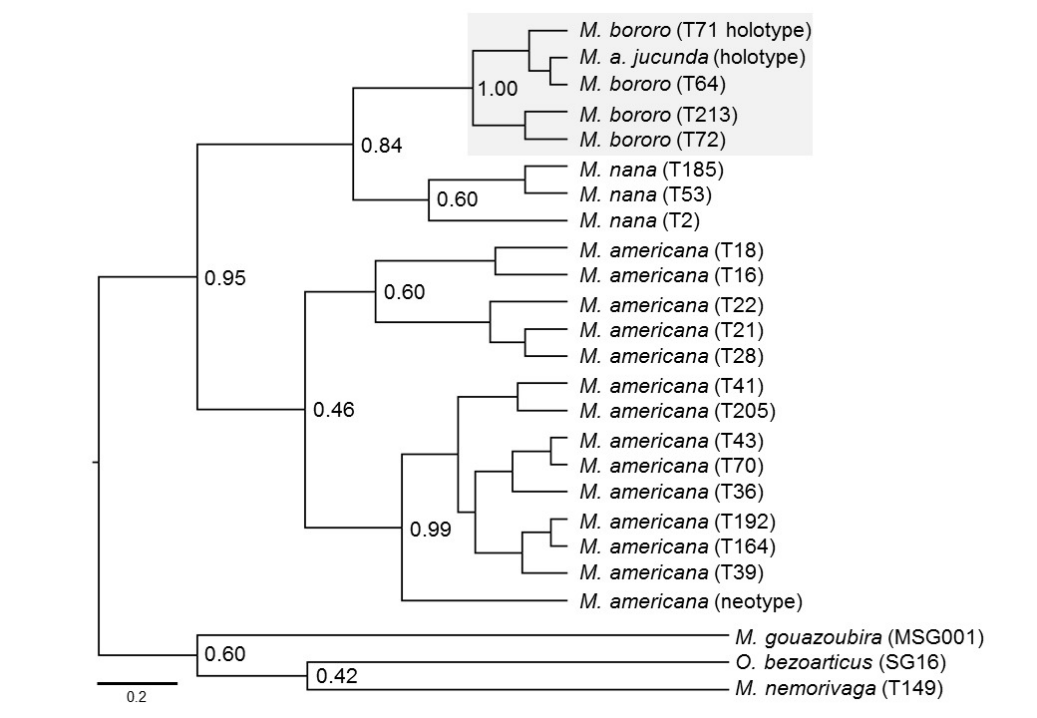
Phylogenetic tree obtained from a Bayesian inference analysis of the unique haplotypes of the mitochondrial cytochrome *b* gene, showing the close phylogenetic relationship between *Mazama bororo* and *Mazama americana jucunda*. The numbers on the nodes of the tree represent the posterior probability values. The sample identification is shown in brackets.

The possibility of obtaining DNA sequences from a type specimen of more than a hundred years old was essential to clarify the taxonomic identity of *M. a. jucunda*. Besides showing that *M. a. jucunda* is not a geographical race, or a subspecies, of *M. americana*, we demonstrate that this taxon is molecularly compatible with the species *M. bororo*, corroborating the similar morphometric and geographic data presented in the description of the two taxa. Therefore, the available name of the small red brocket from the southern Atlantic Forest of Brazil should be *Mazama jucunda* Thomas, 1913, according to the principle of priority (Article 23) of the International Code of Zoological Nomenclature) (ICZN, 1999). 

Genetic resources deposited in museum collections are critically important for scientific research because they allow access to samples that would be difficult or even impossible to obtain today (Schäffer et al., 2017; Tuschhoff et al., 2020). Disputes over the validity of a given taxon can be rapidly solved with comparisons using sequence data from types (Chakrabarty, 2010). In this study, the use of DNA sequences of museum specimens was essential to clarify the taxonomical identity of *M. americana jucunda*, which was known from a specimen collected in 1901.

Johns and Avise (1998) suggested that the cytochrome *b* gene has a high level of congruence within species boundaries, based on classical alpha-taxonomic studies. Our study shows that the cytochrome *b* gene was very informative for elucidating that *M. americana jucunda* represented the same taxon as *M. bororo.*

DNA extracted from museum samples is usually degraded, and only short fragments can usually be amplified (Schäffer et al., 2017). In this context, the low posterior probabilities at the various nodes of the phylogenetic tree are probably related to the small size of the fragment used (224 bp). Nevertheless, the clustering of *M. bororo* and *M. americana jucunda* samples are supported by a posterior probability of 1. The existence of two clades in *M. americana* reflects the existence of different species within the *M. americana*, suggesting the presence of a complex of cryptic species as previously proposed by Duarte et al. (2008) and Cifuentes-Rincón et al. (2020).

Thomas (1913) described the subspecies *M. americana jucunda* based on an immature female from Roça Nova, state of Paraná, in south Brazil, close to localities where *M. bororo* has been recorded (Duarte et al., 2017). Current records suggest that the area of occurrence of the species in this biome is restricted to the Atlantic Forest of the Brazilian states of São Paulo, Paraná, and Santa Catarina, presenting the smallest geographical distribution of the deer species currently described (Vogliotti and Duarte 2010; Duarte *et al.,* 2017). According to Weber and González (2003), the small red brocket deer could be considered being one of the most endangered deer species in the Neotropics, probably due to its endemism in the Atlantic Forest and the intense history of the destruction of this biome. Environmental degradation of the Atlantic Forest is an important threat to *M. jucunda* populations. Likewise, poaching and domestic dogs predation, due to the proximity of human populations, are important threats to the species survival (Vogliotti and Duarte 2010; Duarte *et al.,* 2017). Currently, the species is classified as “Vulnerable” (VU) in the IUCN global assessment (Vogliotti *et al.,* 2016). 

The scientific value of DNA barcode databases would be greatly enhanced if species were also represented by sequences of type specimens (Chakrabarty 2010), as this might mitigate some of the arbitrariness in the correct application of taxonomic names in problematic cases (Johnson et al., 2015; Mutanen et al., 2015). Morphological comparisons should always be part of this process; however, adding molecular markers analyses provide a new dimension for taxonomic research (Chakrabarty, 2010). The unequivocal application of valid names is crucial for all aspects of biodiversity research as well as for monitoring and conservation efforts (Strutzenberger et al., 2012). In this way, changing the name of the small red brocket from *M. bororo* to *M. jucunda* affects the public management and conservation policies of this fragile species in their natural habitat. Finally, we encourage the use of museum DNA of type specimens to provide a more objective and complete comparison with current specimens and consequently offer a more stable taxonomy.
